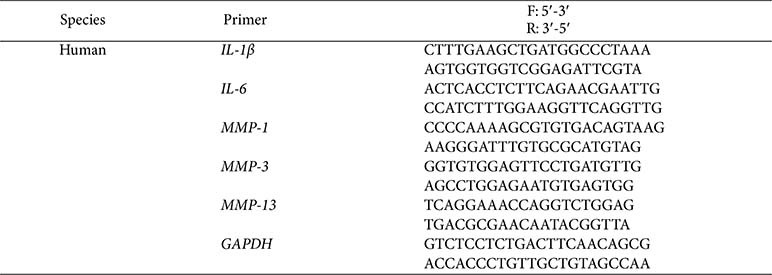# Functional Benefits of Low-Molecular-Weight Collagen Peptide in Achilles Tendon and Medial Collateral Ligament Injuries and Anterior Cruciate Ligament Transection-Induced Osteoarthritis

**DOI:** 10.4014/jmb.2506.06035

**Published:** 2025-09-22

**Authors:** Eunjung Choi, Myunghee Kim, Dae-Hyo Lee, Sehwan Shim, Do-Un Kim, Jeong-Min Hong, Hee-Chul Chung

**Affiliations:** 1 Health Food Research and Development, NEWTREE Co., Ltd., Seoul 05604, Republic of Korea; 2 HLB Biostep Co., Ltd., Incheon, Republic of Korea

**Keywords:** Low-molecular-weight collagen peptide, achilles tendinopathy, anterior cruciate ligament, osteoarthritis, collagen

## Abstract

Musculoskeletal disorders, such as Achilles tendinopathy and anterior cruciate ligament (ACL) injury, can cause serious impairments in physical function and daily life. In particular, ACL rupture increases the risk of osteoarthritis (OA). Collagen maintains the mechanical strength and resilience of tendons and articular cartilage, while low-molecular-weight collagen peptides (LMWCP) support tissue integrity and joint mobility. This study evaluated the efficacy of LMWCP in promoting the repair of Achilles tendon (AT) and medial collateral ligament (MCL), and preventing ACL transection (ACLT)-induced OA. In animal models, LMWCP was administered following AT and MCL injuries, as well as ACLT. LMWCP treatment in the ACLT rabbit model significantly improved cartilage integrity, reducing surface damage, proteoglycan loss, and OARSI scores. It also inhibited subchondral bone deterioration and osteophyte formation, restoring bone volume fraction, as shown by Micro-CT analysis. Moreover, LMWCP administration increased type II collagen expression while decreasing that of matrix metalloproteinases (MMPs) in cartilage tissue, and decreased inflammatory cytokine concentrations in the synovial fluid. In IL-1β–stimulated human chondrocytes, LMWCP suppressed inflammatory cytokines and MMP expression, and increased hydroxyproline content, indicating reduced collagen degradation. In the AT and MCL defect model, LMWCP promoted tissue regeneration by improving fiber arrangement, rounding of nuclei, angiogenesis, and cell density and promoting collagen deposition. These findings suggest that LMWCP may enhance joint function and tendon healing through modulation of inflammation and collagen remodeling, representing a functional ingredient for promoting tendon and ligament repair and attenuating ACLT-induced OA progression.

## Introduction

Physical activity is widely known to be beneficial for preventing musculoskeletal disorders. However, intense physical activity can increase the risk of injury, including tendon, ligament, and joint damage, which can affect joint structures, such as the articular cartilage [1]. Musculoskeletal disorders, including Achilles tendinopathy and anterior cruciate ligament (ACL) injury, are common conditions that significantly reduce mobility and quality of life

Achilles tendinopathy is one of the most common injuries related to physical exercise [2]. The Achilles tendon (AT) is one of the longest tendons in the body and connects the heel bone to the calf muscles. It is subjected to excessive stress due to repetitive, intense activities, such as running, jumping, and landing. These activities can cause structural changes and biomechanical weakening of the tendon, leading to rupture [3]. AT ruptures occur at an estimated rate of 10 per 100,000 people annually, making them a significant concern in orthopedics and sports medicine. Treatment of Achilles tendinopathy is challenging, and even after surgical intervention, recurrence rates remain high, reaching 44% [4]. Achilles tendinopathy is particularly problematic because it often has a long latent period, making early diagnosis difficult [5]. Consequently, Achilles tendinopathy is frequently underestimated and mismanaged. Athletes with AT ruptures typically require a long recovery period, which can have significant psychological and physiological impacts [6-8].

ACL injuries are among the most frequently reported knee injuries, with a particularly high incidence among athletes and physically active individuals [9]. The knee joint is the most commonly injured site because it connects the two longest bones in the body and bears the entire body weight during activities, such as walking, running, or jumping. The reported frequency of ACL rupture (ACLR) in the general population is approximately 0.8 per 1,000 individuals [10]. ACLR is particularly significant because it is the primary cause of osteoarthritis (OA) in young patients. Although most patients with ACLR undergo ACL reconstruction, 50% of patients who undergo this treatment develop OA. Given the high frequency of ACL ruptures among young individuals, managing the risk of OA following ACL injury is essential [11].

Collagen constitutes approximately 65–80% of the dry weight of tendons, and collagen crosslinks play a crucial role in helping tendons resist high impact stresses and shear forces [12]. Collagen is also a primary extracellular matrix (ECM) component of cartilage. It reportedly inhibits bone breakdown and alleviates pain symptoms associated with degenerative joint conditions [13]. Consequently, collagen can be considered a safe and beneficial functional ingredient for maintaining tendon and joint health [14, 15]. Clinically, oral supplementation with collagen peptides increases skin moisture after 8 weeks of intake and dermal collagen density after 4 weeks [16]. Additionally, low-molecular-weight collagen peptides (LMWCPs) exhibit higher amino acid digestion and absorption rates in the intestine [17], and consumers prefer low-molecular-weight collagen to other collagen types [18].

This study aimed to comprehensively evaluate the effects of LMWCPs on musculoskeletal injuries associated with physical activity, with a particular focus on AT injuries and knee cartilage injuries following ACLR. Toward this, we evaluated the efficacy of LMWCPs in promoting tendon and ligament repair and attenuating OA progression induced by ACL injury. We focused on whether LMWCP administration suppressed inflammatory cytokine expression, decreased matrix metalloproteinase (MMP) activity, and increased collagen production.

## Materials and Methods

### Test Material

In this study, we used LMWCP supplied by NEWTREE, Co., Ltd. LMWCP is a collagen hydrolysate prepared using the skin of *Pangasius hypophthalmus*. It contains Gly-Pro-Hyp (about 3%) and tripeptide (over 15% w/w). LMWCP was dissolved in distilled water before use.

### Experimental Cell Line

The SW 1353 chondrocyte cell line was obtained from the American Type Culture Collection. All reagents used for cell culture were purchased from Gibco (USA). Cells were cultured in Dulbecco’s modified Eagle’s medium supplemented with 10% fetal bovine serum and 1% antibiotic-antimycotic solution. The cell cultures were maintained at 37°C in a humidified incubator with 5% CO_2_.

### Rabbit ACL Transection (ACLT)-Induced OA Model

Twenty-four New Zealand white rabbits (≥2.5 kg; male) were purchased from Saeronbio (Republic of Korea) and maintained in an animal facility with controlled conditions (temperature, 23°C ± 3°C; relative humidity, 55%± 15%; ventilation frequency, 10–20 times/h; lighting time, 12 h [8 am–8 pm]; and illuminance, 150–300 Lux [IACUC Approval No. 23-HB-0486]). After one week of acclimation, the rabbits underwent ACLT on the right joint to induce OA. Briefly, the animals were anesthetized, and the hair around the right knee was removed using clippers. The surgical site was extensively disinfected with povidone and 70% alcohol, and the skin was incised. The surrounding tissue was bluntly incised to expose the articular surface of the distal right femur. The ACL was incised with surgical scissors, and the wound was sutured with 4-0 nylon. After OA induction, antibiotics (cephradine) and analgesics (tramadol) were administered for three days to suppress the inflammation caused by surgery [19, 20]. One week after the surgery, the rabbits were divided into three groups (*n* = 8). The sham and ACLT groups were orally administered distilled water or LMWCP (200 mg/kg body weight/day) for 12 weeks. During the experimental period, rabbits had free access to food and water. After the experiment, the rabbits were fasted for 12 h, after which the synovial fluid was collected, and the animals were euthanized. Tissues from the ACLT site were fixed in 10% neutral-buffered formalin solution.

### AT and Medial Collateral Ligament (MCL) Defect Model

Nine New Zealand white rabbits (≥2.5 kg; male) were purchased from Saeronbio (Republic of Korea) and maintained in an animal facility with controlled conditions (temperature, 23°C ± 3°C; relative humidity, 55% ± 15%; ventilation frequency, 10–20 times/h; lighting time, 12 h [8 am–8 pm]; and illuminance, 150–300 Lux [IACUC Approval No. 24-HB-0114]). After one week of acclimation, defect surgery was performed on the AT and MCL on both sides to establish the tendon defect model. Briefly, after the animals were anesthetized, the periphery of the AT and MCL on both sides was epilated using clippers. The surgical site was widely disinfected with povidone and 70% alcohol, and the skin around the inner knee of the right hind limb was incised, followed by incision of the skin around the AT of the right hind limb. The surrounding tissue was subjected to blunt dissection to expose the AT, and a defect was created in the AT using a 2-mm biopsy punch. The skin was then sutured using 4-0 nylon. The surrounding tissue was subjected to blunt dissection to expose the MCL, and a defect was created in the MCL using a 2-mm biopsy punch. The same surgical procedure was repeated for the left hindlimb. After surgery, the rabbits were divided into three groups (*n* = 3). The sham and defect groups were orally administered distilled water or LMWCP (200 mg/kg body weight/day) for six weeks. The rabbits had free access to food and water during the experiments. At the end of the experiment, the rabbits were fasted for 12 h before euthanasia. The defect site tissues were fixed in 10% neutral-buffered formalin solution.

### Reverse Transcription Quantitative PCR (RT-qPCR) Analysis

SW 1353 cells were treated with IL-1β and various concentrations of test samples (100, 300, 500 and 1,000 μg/ml) for 24 h. Total RNA was extracted from each experimental group using TRIzol reagent. The extracted RNA was quantified, and an equal amount of RNA was used for reverse transcription. The mRNA expression levels of target genes, including *MMP-1*, *MMP-3*, *MMP-13*, *IL-6*, and *IL-1β*, were determined using RT-qPCR with specific primers (Table 1) and SYBR Green fluorescence dye.

### Hydroxyproline Assay

Following a 24 h treatment with IL-1β and different concentrations of test samples, the total hydroxyproline content was assessed using a Hydroxyproline Assay Kit (BM HYP-100, BIOMAX, Republic of Korea).

### Micro-Computed Tomography (CT)

The subchondral bone microarchitectures of the femur and tibia were measured using a micro-CT system (Scanco Medical, Switzerland). The measurement parameters were as follows: eddy size: 60 μm, energy source: 55 kVp, intensity: 145 μA, integration time: 150 ms. The evaluation of the knee joint was performed by measuring and analyzing the structural elements and bone density of the femur and tibia from cross-sectional images acquired through the program. Quantitative analyses included trabecular bone volume/total volume (BV/TV), trabecular bone surface area/bone volume (BS/BV), trabecular bone number (TbN), trabecular bone thickness (TbTh), and trabecular separation (TbSp).

### Biochemical Analyses

IL-1β and IL-6 concentrations in synovial fluid were analyzed using commercially available ELISA kits (IL-1β, RnD Systems, RLB00-1, USA; IL-6, RnD Systems, R6000B, USA) according to the manufacturer’s instructions.

### Histological Analyses

The fixed tissues were decalcified using a commercially available unbuffered 10% EDTA decalcification solution and embedded in paraffin. Paraffin-embedded tissues were sectioned at 4 μm thickness and stained with hematoxylin and eosin (H&E) or Safranin O according to standard protocols. OA progression was assessed using the Osteoarthritis Research Society International (OARSI) method as described previously [19, 20], and tendon defect was assessed using histological scoring as described by Shu *et al*. [21]. The scoring criteria were based on the parameters suggested in their study on atelocollagen-mediated ligament and tendon healing. Each parameter, including fiber structure, fiber arrangement, rounding of nuclei, angiogenesis, cell density, and collagen stainability, was evaluated on a scale of 0 to 3 (0 = normal, 3 = severely abnormal).

For immunohistochemical staining, sections were stained with type I collagen (Abcam, ab260043, USA), type II collagen (Abcam, ab188570, USA), and MMP13 (Invitrogen, MA5-14238, USA) antibodies according to the standard protocol. Quantitative analysis of staining was performed using an image analysis software (Zen 2.3 Blue Edition, Carl Zeiss, Germany).

### Statistical Analyses

All *in vitro* experiments were repeated at least three times, and the results were expressed as the mean ± SD using SPSS software (ver. 12.0, SPSS Inc., USA). Differences were analyzed using one-way analysis of variance followed by Duncan’s test. Differences with *p*-value < 0.05 were considered statistically significant. For *in vivo* experiments, data were assumed to follow a normal distribution, and statistical significance between groups was assessed using the Student’s *t*-test. Statistical analysis was performed using Prism 10.2.3 (GraphPad Software Inc., USA), and differences with *p*-value < 0.05 were considered statistically significant. The statistical methods were selected based on the study design, number of groups being compared, and assumptions of normality and homogeneity of variance.

## Results

### LMWCP Improved Cartilage Damage associated with OA in the ACLT Rabbit Model

**Fig. 1:**
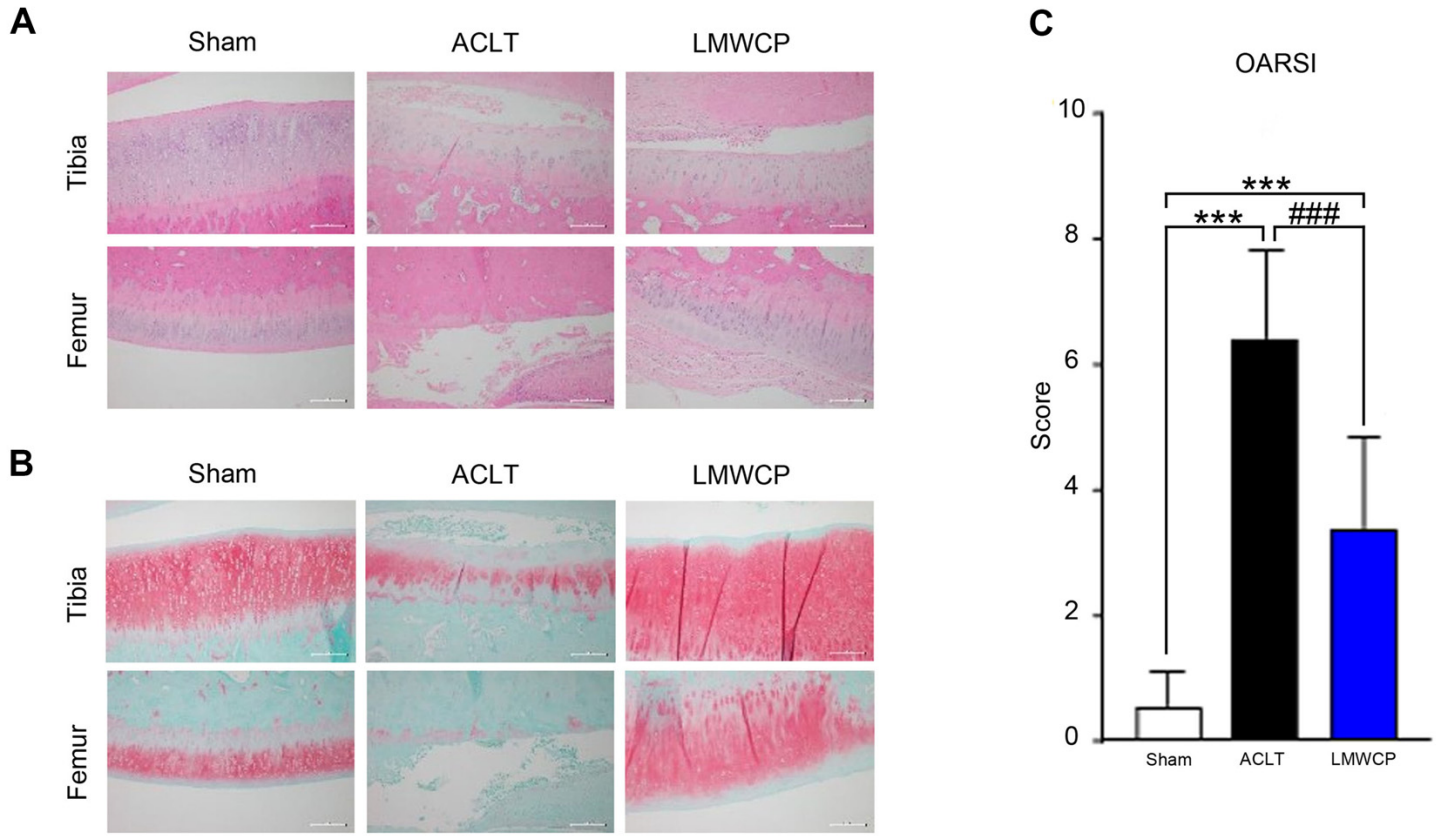
Low-molecular-weight collagen peptide (LMWCP) improved osteoarthritis-associated cartilage damage in an ACLT model. After ACLT or sham surgery, rabbits were orally administered vehicle or LMWCP (200 mg/kg) once daily for 12 weeks. (**A**) Representative hematoxylin and eosin (H&E) staining of the tibia and femur (magnification, ×100). (**B**) Representative Safranin O staining images of the tibia and femur (magnification ×100). (**C**) Quantitative analysis of cartilage degeneration based on OARSI scores. Data represent mean ± standard deviation (*n* = 8). Statistical analysis was performed using *t*-test. ***p < 0.001, compared with sham group. ###*p* < 0.001, compared with ACLT group.

To investigate its cartilage-protective effect, LMWCP was administered orally to ACLT-induced OA rabbits for 12 weeks. Cartilage damage was assessed by H&E and Safranin O staining, which confirmed cartilage degeneration during OA progression [22]. H&E staining (Fig. 1A) revealed that the cartilage surface was damaged and hypocellular following OA induction. Safranin O staining showed that the ACLT group had severe proteoglycan loss compared to the sham group. However, the cartilage surface of the rabbits in the LMWCP group was smoother than that of the rabbits in the ACLT group (Fig. 1B). Moreover, hypocellularity due to OA in the ACLT group was alleviated by LMWCP. In addition, proteoglycan loss was significantly less severe in the LMWCP group than in the ACLT group. The OARSI score (Fig. 1C), which increased after OA induction, was reduced by LMWCP. These results suggest that LMWCP exerts a protective effect on cartilage in this OA model.

### LMWCP Inhibited OA-Related Subchondral Bone Damage in the ACLT Rabbit Model

**Fig. 2:**
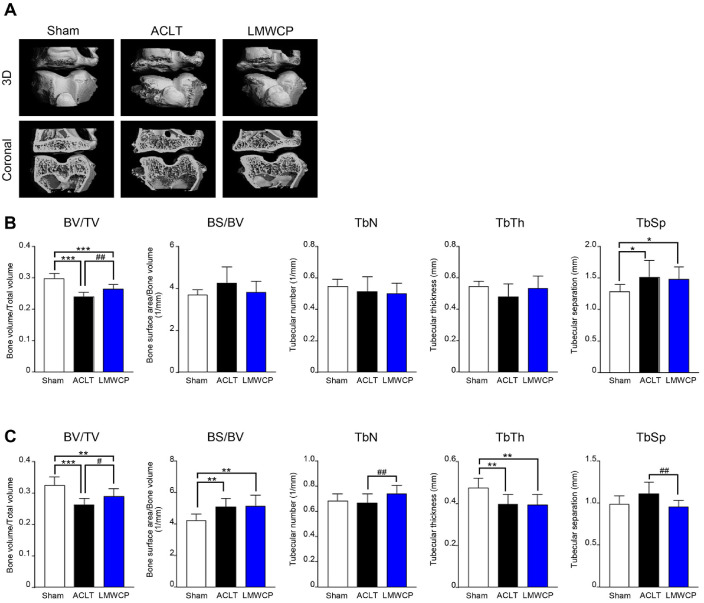
Low-molecular-weight collagen peptide (LMWCP) suppressed osteoarthritis-associated subchondral bone damage in a rabbit ACLT model. Rabbits underwent ACLT or sham surgery and were orally administered vehicle or LMWCP (200 mg/kg) once daily for 12 weeks. (**A**) Representative micro-computed tomography (CT) images of subchondral bone. Quantitative micro-CT analysis of subchondral bone in the tibia (**B**) and femur (**C**). Trabecular bone volume/total volume (BV/TV), trabecular bone surface area/bone volume (BS/BV), trabecular bone number (TbN), trabecular bone thickness (TbTh), and trabecular separation (TbSp). Data represent mean ± standard deviation (*n* = 8). Statistical analysis was performed using *t*-test. *p < 0.05, **p < 0.01 and ***p < 0.001 compared with sham group. #*p* < 0.05 and ##*p* < 0.01 compared with ACLT group.

To investigate the subchondral bone damage that occurs after cartilage damage in OA, we analyzed morphological and microstructural changes in the subchondral bone using micro-CT. The number of osteophytes, which severely increased due to bone damage in the ACLT group, decreased in the LMWCP group (Fig. 2A). In addition, micro-CT analysis demonstrated a significant reduction in bone volume fraction (BV/TV) in both the tibial and femoral subchondral bones of the ACLT group compared to that in the sham group (Fig. 2B and 2C). Notably, LMWCP treatment markedly restored BV/TV in both regions. Furthermore, in the femoral subchondral bone, LMWCP treatment resulted in an increased TbN and a decreased TbSp, indicating that LMWCP may help preserve subchondral bone microarchitecture.

### Effect of LMWCP on IL-1β-Stimulated Human Chondrocytes and Synovial Fluid Proinflammatory Cytokines in the Rabbit ACLT Model

**Fig. 3:**
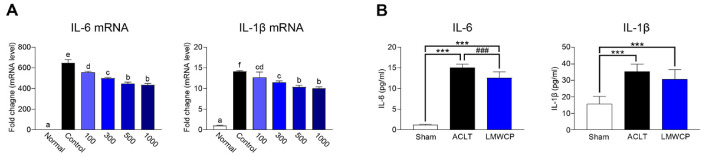
Low-molecular-weight collagen peptide (LMWCP) inhibits proinflammatory cytokines in IL-1β- stimulated human chondrocytes and synovial fluid from rabbit ACLT model. (**A**) mRNA expression of *IL-6* and *IL-1β* was quantified by qRT-PCR to evaluate the inhibitory effect of LMWCP on IL-1β-induced proinflammatory cytokines. Data are presented as the mean ± SD (*n* = 3) (**B**) Protein levels of IL-6 and IL-1β were measured by ELISA in synovial fluid from rabbits that underwent either ACLT or sham surgery and were treated with LMWCP (200 mg/kg) or vehicle via oral gavage once daily for 12 weeks. Data represent mean ± standard deviation (*n* = 8). Statistical analysis was performed one-way ANOVA followed by Duncan’s test for *in vitro* data and using t-test for *in vivo* data. a~f) The statistical significance level of the average value for each experimental group is indicated by each subgroup for *p* < 0.05. ***p < 0.001, compared with sham group. ###*p* < 0.001, compared with ACLT group.

To investigate the anti-inflammatory effect of LMWCP, the mRNA expression levels of *IL-1β* and *IL-6* in human chondrocytes stimulated with IL-1β were analyzed using qRT-PCR. As shown in Fig. 3A, IL-1β stimulation increased the expression of *IL-6* and *IL-1β* compared with that in the normal group, whereas LMWCP significantly reduced the expression levels of both cytokines. These results suggested that LMWCP attenuates IL-1β-induced inflammatory gene expression, which may be involved in modulating the inflammatory response associated with OA progression.

To verify the protein-level effect of LMWCP in ACLT models, synovial concentrations of IL-1β and IL-6, the major inflammatory cytokines associated with OA, were measured by ELISA. As shown in Fig. 3B, LMWCP administration caused a significant decrease in the levels of IL-6 and induced a decreasing trend in IL-1β concentrations. These results suggested that LMWCP effectively inhibits the inflammatory response by regulating the expression of key cytokines at both the transcriptional and protein levels. This supports the potential of LMWCP in the management of OA-associated inflammation, especially in IL-1β-stimulated chondrocytes and ACLT animal models.

### Effect of LMWCP on Collagen Preservation and MMP Expression in IL-1β-Stimulated Human Chondrocytes and the Rabbit ACLT Model

**Fig. 4:**
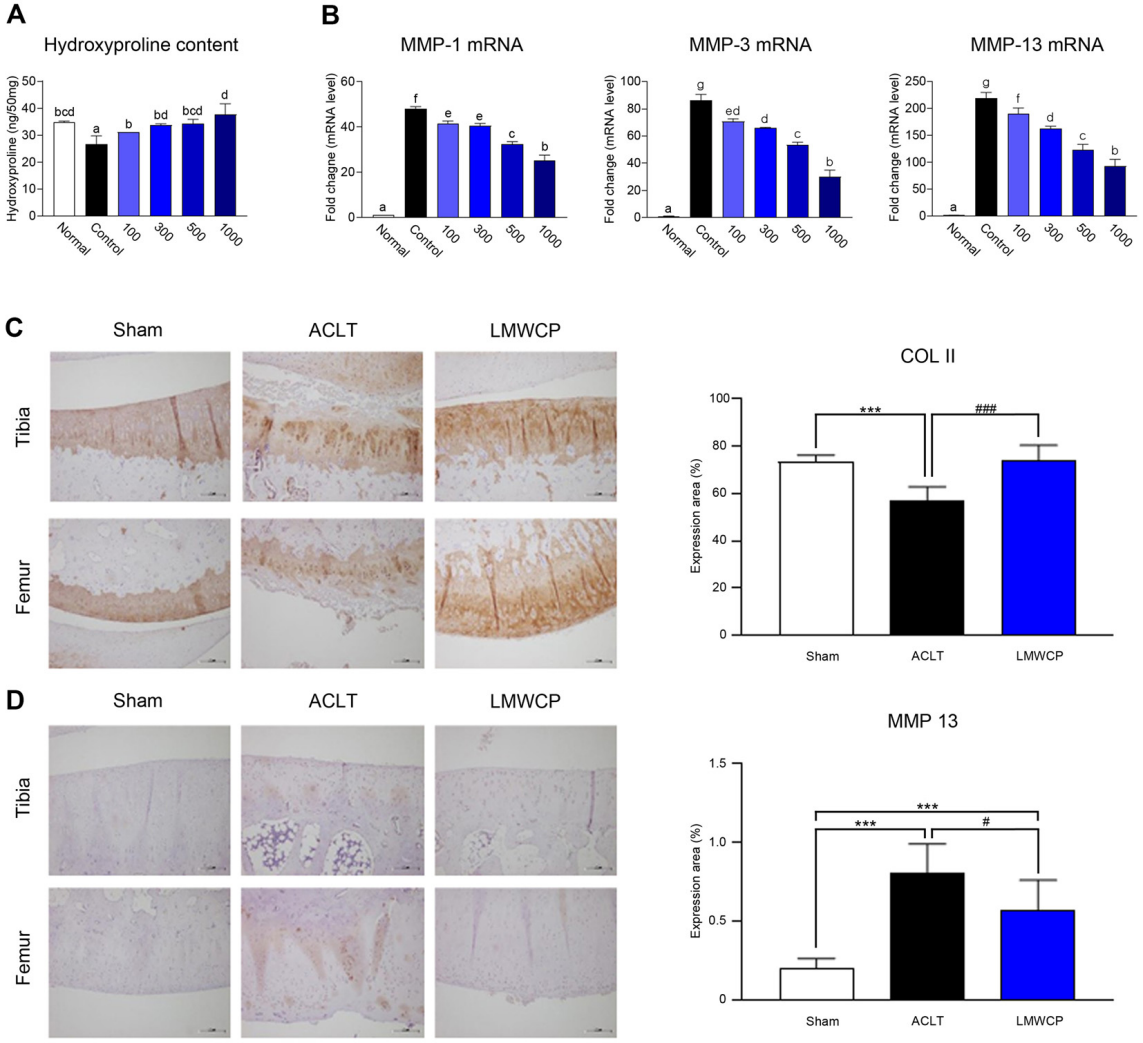
Effect of low-molecular-weight collagen peptide (LMWCP) on collagen preservation in human chondrocytes and rabbit cartilage of ACLT model. (**A**) Collagen degradation was assessed by measuring hydroxyproline content in IL- 1β (0.1 ng/ml)-stimulated SW1353 chondrocytes treated with different concentrations of LMWCP (100–1,000 μg/ml). Data are presented as the mean ± SD (*n* = 3) (**B**) mRNA expression of *MMP-1*, *MMP-3*, and *MMP-13* was quantified by qRT-PCR to evaluate the inhibitory effect of LMWCP on IL-1β-induced MMP upregulation. Data are presented as the mean ± SD (*n* = 3) (**C**) Representative immunohistochemical images of cartilage (magnification, ×100). Quantitative analysis of type II collagen (COL II). (**D**) Representative immunohistochemical images of cartilage (magnification, ×100). Quantitative analysis of MMP- 13 staining. Data represent mean ± standard deviation (*n* = 8). Statistical analysis was performed using one-way ANOVA followed by Duncan’s test for *in vitro* data and using *t*-test for *in vivo* data. a~g) The statistical significance level of the average value for each experimental group is indicated by each subgroup for *p* < 0.05. ***p < 0.001, compared with sham group. #*p* < 0.05 and ###*p* < 0.001 compared with ACLT group.

LMWCP significantly increased the hydroxyproline content, indicating reduced collagen degradation in human chondrocytes stimulated with IL-1β (Fig. 4A). Additionally, LMWCP treatment decreased *MMP-1*, *MMP-3*, and *MMP-13* mRNA levels in a dose-dependent manner, which were upregulated by IL-1β stimulation (Fig. 4B). These findings suggest that LMWCP protects the cartilage by inhibiting MMP expression and preserving collagen integrity. In addition, we confirmed the protective effects of LMWCP in ACLT models. To elucidate the molecular mechanism of LMWCP, the expression of type II collagen and MMP-13 in the cartilage was analyzed using immunohistochemistry (Fig. 4C and 4D). LMWCP administration significantly upregulated type II collagen expression in cartilage tissue, which had been reduced by OA. In the LMWCP group, a significant decrease in MMP-13 expression was observed compared with that in the ACLT group, indicating that LMWCP may exert a protective effect by regulating ECM synthesis and degradation in cartilage tissue.

### Effect of LMWCP on Tendon Healing and Collagen Preservation in AT and MCL Defect Model

**Fig. 5:**
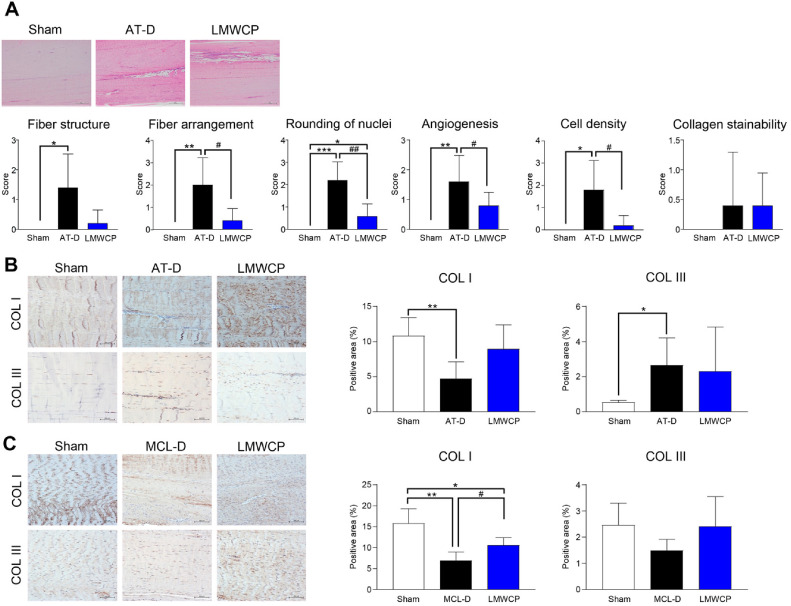
LMWCP enhances tendon healing and collagen preservation in the medial collateral ligament and Achilles tendon (AT) defect model. Rabbits underwent Achilles tendon injury and received either vehicle or LMWCP (200 mg/kg) via oral gavage once daily for six weeks. (**A**) Representative hematoxylin and eosin (H&E)-stained images of the areas in the AT defect (AT-D) (magnification ×100). Fiber structure, fiber arrangement, rounding of nuclei, angiogenesis, cell density, and collagen stainability were assessed in the AT. (**B, C**) Representative immunohistochemical images of type I collagen (COL I) and type III collagen (COL III) staining in the AT defect area (**B**) and medial collateral ligament defect area (**C**) (magnification ×100). The proportion of collagen–positive areas was quantified. Data represent mean ± standard deviation (*n* = 5). Statistical analysis was performed using *t*-test. *p < 0.05, **p < 0.01 and ***p < 0.001 compared with sham group. #*p* < 0.05 and ##*p* < 0.01 compared with AT-D and MCL-D group.

To evaluate the effects of LMWCP on tendon healing and collagen preservation, histopathological and immunohistochemical analyses were performed on the AT and MCL defect model. As presented in Fig. 5A, LMWCP treatment led to a reduction in nuclear rounding, fiber arrangement, angiogenesis, and cell density in the AT tissue. Furthermore, immunohistochemical analysis showed that type I collagen expression was significantly reduced in the AT and MCL defect model. LMWCP was shown to prevent the decrease of type I collagen in the AT and significantly increased type I collagen expression in the MCL (Fig. 5B and 5C). In addition, type III collagen expression showed a decreasing trend in the MCL defect model, which was partially restored by LMWCP treatment (Fig. 5C). These findings indicate that LMWCP regulates collagen composition during tendon healing. Taken together, these results demonstrate that LMWCP exerts protective effects on tendon healing by improving fiber structure and preserving collagen integrity in the MCL and AT defect model.

## Discussion

As the AT and knee cartilage are highly susceptible to injury during sports and physical activity, effective post-injury management strategies are essential for functional recovery and prevention of long-term disability. This study evaluated the beneficial effects of LMWCP in models of AT and MCL defect and ACLT-induced OA. The results showed that LMWCP exerted significant protective effects on joint and tendon health by reducing inflammation, preserving collagen integrity, and improving tissue architecture. These results suggest that LMWCP may serve as a beneficial nutritional component for maintaining musculoskeletal health, especially in diseases involving tendon and cartilage damage. One of the major findings of this study is that LMWCP effectively ameliorated cartilage damage in the ACLT model. Histological analysis showed that LMWCP treatment improved cartilage integrity by preserving cartilage structure, reducing proteoglycan loss, and reducing OARSI scores. These observations are consistent with those of a previous study showing that collagen supplementation supports cartilage maintenance and regeneration [23]. In addition, LMWCP exhibited protective effects against subchondral bone damage during OA progression. Micro-CT analysis revealed that LMWCP administration increased the BV/TV of the tibia. In the femur, LMWCP also increased BV/TV and TbN, while decreasing the TbSp. Considering that subchondral bone remodeling plays an important role in OA pathogenesis, these results suggest that LMWCP supplementation may help maintain bone integrity and alleviate OA progression.

Inflammation is the major cause of cartilage degradation in OA [24]. *In vitro* and *in vivo* experiments showed that LMWCP significantly reduced the expression of inflammatory cytokines, including IL-6 and IL-1β, in IL-1β-stimulated human chondrocytes and in ACLT rabbit synovial fluid. The dose-dependent inhibition of these cytokines by LMWCP suggests a potent anti-inflammatory action, which may contribute to its cartilage-protective action. These results are consistent with reports that collagen peptides regulate inflammatory pathways and reduce cytokine levels in OA [25].

MMPs play a critical role in ECM degradation during OA progression [26]. The results of this study showed that the expression of MMP-1, MMP-3, and MMP-13 was significantly reduced in IL-1β-stimulated chondrocytes following LMWCP administration, suggesting that LMWCP plays an important role in the maintenance of cartilage structure. Additionally, immunohistochemical analysis revealed that LMWCP administration increased the expression of type II collagen and decreased the expression of MMP-13 in cartilage. Type II collagen, a major component of the cartilage matrix, helps form complex extracellular scaffolds to support mechanical function, maintain homeostasis, and anchor chondrocytes and signaling molecules [27]. Its degradation and reduction are hallmark pathological features frequently observed in OA cartilage [28]. These results suggest that LMWCP has potential as a functional ingredient to maintain joint health and prevent OA progression by attenuating ECM degradation and maintaining collagen integrity.

In addition to its chondroprotective effects, LMWCP significantly improved tendon and ligament healing in an AT and MCL defect model. Histopathological evaluation showed that LMWCP reduced fiber order, nuclear circularization, neovascularization, and cell density. Further, immunohistochemical analysis showed that collagen type I expression increased in LMWCP-treated ligaments, suggesting that LMWCP promoted collagen synthesis and remodeling during tendon repair. A tendency for recovery of type III collagen expression was also observed in the MCL model. Type I collagen constitutes approximately 80% of the dry weight of tendons and ligaments and is primarily responsible for the core strength of these tissues [29]. In contrast, type III collagen intercalates into type I collagen fibrils, resulting in smaller and less organized fibrils [30]. These findings are consistent with those of previous studies emphasizing the importance of collagen supplementation in ECM stabilization and tissue repair [31, 32]. Moreover, these observations are consistent with previous studies showing that bioactive peptides can alleviate inflammatory responses, while promoting collagen synthesis [25]. The beneficial effects of LMWCP may be related to its high bioavailability and potential to contribute to the health of connective tissues, which is supported by previous studies showing that LMWCPs are efficiently absorbed and utilized in tissue scaffolds [33]. Therefore, based on the histopathological and immunohistochemical analyses, LMWCP is predicted to improve tendon and ligament healing and preserve collagen in AT and MCL defect models.

Overall, these results are consistent with previous studies showing that collagen peptides contribute to tendon healing by increasing fibroblast proliferation, ECM synthesis, and collagen deposition [31, 34, 35]. As Achilles tendonitis and tendon ruptures are difficult to treat and have a high rate of recurrence, LMWCP supplementation may constitute a promising noninvasive strategy to improve tendon recovery and functional outcomes.

One limitation of this study is the relatively short duration of the *in vivo* experiment. Due to the limited observation period, the results may not fully reflect the long-term efficacy, safety, or potential delayed effects of the treatment. Thus, longer follow-up studies are needed to verify and further develop the findings of this study.

In summary, this study demonstrated that LMWCP exerts significant protective effects on joint and tendon health by mitigating inflammation, preserving collagen integrity, and improving tissue structure. LMWCP holds promise as a functional ingredient for promoting AT injury healing and preventing OA progression following ACL injury.

## Figures and Tables

**Table 1:** RT-qPCR primer sequences used in this study.